# Cochlear implants in adults with partial deafness: subjective benefits but associated psychological distress

**DOI:** 10.1007/s00405-020-06199-x

**Published:** 2020-07-15

**Authors:** Joanna Kobosko, W. Wiktor Jedrzejczak, Anna Barej, Agnieszka Pankowska, Anna Geremek-Samsonowicz, Henryk Skarzynski

**Affiliations:** 1 Institute of Physiology and Pathology of Hearing, Warsaw, Poland; 2 World Hearing Center, ul. Mokra 17, Kajetany, 05-830 Nadarzyn, Poland

**Keywords:** Cochlear implants, Hearing-related quality of life, Psychological distress, CI satisfaction level

## Abstract

**Purpose:**

The present study investigated adults with partial deafness (PD) and asked them to rate the benefits of their cochlear implant (CI), their general level of satisfaction with it, and their level of psychological distress. Of particular interest was the role of gender.

**Methods:**

The study comprised 71 participants (41 females) with PD who had been provided with a CI. The *Nijmegen Cochlear Implant Questionnaire* (NCIQ) was used to assess the benefits of their CI. Satisfaction with their CI was measured using a visual analog scale. The severity of mental distress was assessed with the *General Health Questionnaire* (GHQ-28).

**Results:**

On various NCIQ scales, the average benefits of a CI were rated at 66%. Females gave a lower rating than males. The mental distress experienced by the group was significantly higher than in the general population. Females had more severe symptoms of anxiety and insomnia than males. There was a significant relationship between psychological distress and CI benefit, but only in females. Besides general distress, the most affected spheres were related to psychosocial functioning—“self-esteem”, “activity limitation”, and “social interaction”. Contrary to expectations, there was no relationship between mental distress and CI satisfaction.

**Conclusions:**

The perceived benefits of a CI in subjects with PD relate mostly to the level of mental distress, although gender is an important factor. For females, their emotional state affects how beneficial their CI is perceived. Due to the higher levels of mental distress, females tend to need more psychological intervention and support.

## Introduction

When users subjectively rate the effectiveness of their cochlear implants (CIs), two aspects need to be considered: the actual perceived benefits of the CI and the general level of satisfaction. These two factors are good indicators of the effectiveness of a CI in overcoming hearing loss [1–5]. The benefits relate to how highly the user rates the performance of the device, expressed in terms of sound clarity (especially of speech), ease of communication, and facilitation of social interactions [6]. Satisfaction with a CI can be understood “as the subjective state of a CI user that reflects the overall feeling of benefit, audiological and non-audiological, attributed to their CI, including quality of life and general psychological well-being” [7].

Such assessments are usually made using specific tools, e.g., the *Nijmegen Cochlear Implant Questionnaire* (NCIQ) [2, 4, 6] or the *Satisfaction with Amplification in Daily Life* (SADL) questionnaire [3], but visual analog scales (VAS) can also be used [7]. In general, research shows that self-reported levels of satisfaction with a CI appears to be high to very high (whether the users had pre- or post-lingual deafness), reaching about 80% [3, 7, 8]. The benefits of a CI assessed using the NCIQ vary, and range from 50 to 80% [1, 4, 6, 8–12].

Another trend in research on assessing the benefits of CIs and satisfaction with them is the crucial link to objective test results. Traditionally, these include clinical tests of auditory perception, auditory recognition, and understanding of speech [9, 13, 14]. Low to moderate correlations have been found between the subjective assessment of the benefits of a CI and its objective measurement [11, 13, 14], but when it comes to the link between satisfaction with a CI and the results of objective tests, the correlation is small or non-existent [7].

Partial deafness (PD) is a type of hearing loss in which the patient fails to hear sounds at high frequencies, but at low frequencies they hear quite well, or have a degree of loss that can be adequately compensated using a hearing aid. The best outcomes of treating PD usually come by using the CI for high-frequency electrical stimulation, and using natural hearing or a hearing aid (HA) for the low frequencies [15]. Patients with PD often complain of a lack of speech understanding [4], frequently experiencing problems with social interactions, including with their family and in the workplace. Sometimes, they try to hide their hearing problem [4, 16, 17]. PD often presents a sort of paradox in that often the patient can hear, but does not understand, or only partially understands, verbal messages. Cognitive dissonance can also be experienced as an awkward reaction to other people, who can in turn be confused. Deaf and hard of hearing (DHH) people, including those with PD, constantly feel a strain in everyday life, giving rise to tension, stress, uncertainty, or fatigue [17]. This often leads to stigma, so the social context of DHH people should not be overlooked [16, 18].

Studies into mental distress of CI users and the subjective assessment of the benefits and satisfaction levels with their CI are still scarce. It is already known that, compared to the general population, DHH people without a CI experience greater mental distress, including symptoms of depression and anxiety, regardless of the etiology of their deafness or the degree of hearing loss [16, 17, 19–24]. DHH females have a higher level of mental distress [19, 23], including depression [22, 25] and anxiety [26], than DHH males, a gender relationship also known for the general population [22, 27]. In fact, research on CI users is increasingly finding that mental distress, as well as related symptoms of depression, anxiety, somatic symptoms, and disorders in social functioning, is at similar levels as in the general population [7, 28]. Research points to those CI users who have a lower level of distress before receiving their CI eventually having better objective results in terms of speech outcomes [21, 29]. Interestingly, two studies indicate that, in post-lingually deaf subjects at least, there is a negative relationship between the subjective assessment of the benefits of a CI and symptoms of depression and anxiety. In other words, patients who have a lower perception of CI benefits have at the same time a higher intensity of depression and anxiety [7, 29].

Given this complex picture, the aim of the present study was to assess the benefits and satisfaction of CIs perceived by persons with PD, as well as to assess their mental distress, in particular to examine the relationship between these variables while taking the role of gender into account.

## Materials and methods

### Participants

Persons qualified for the study met the following criteria: (a) were diagnosed as having partial deafness (PD), defined as having severe to profound hearing loss at frequencies above 1 kHz and with 70-dB HL threshold or better from 0.25 to 1 kHz; (b) provided with one CI in PD ear after 18 years of age, which they had used for at least one year (some had used only a CI, while others had an HA in the unimplanted ear); (c) had at least partial postoperative hearing preservation (HP) according to the classification of Skarzynski et al. [30]; this included subjects with complete HP (pure tone average (PTA) change after receiving a CI of 0–10-dB HL) and partial HP (PTA change of 10–30-dB HL); (d) had aided speech perception scores of 60% in quiet and 40% in noise (or better) for Polish monosyllabic words tests [31]; (e) onset of deafness could either have been pre-lingual or post-lingual; (f) their age was 18–60 years; and (g) they communicated aurally/verbally (not by sign language). The characteristics of the respondents (gender, type of deafness, and cochlear implants) are given in Table 1. Females and males differed only in marital or partner status.

**Table 1:** Sociodemographic data on study participants and on the duration of deafness and CI experience, including CI satisfaction (sat-VAS)

	All participants	Female	Male	Statistical test (female vs male)
*N*	71	41	30	
Age (years)				
Mean (SD)	41.8 (11.9)	43 (11.85)	40.1 (11.97)	*t*(69) = 1.001; n.s.
Range	22–60	23–60	22–60	
Educational status				
Primary or secondary (%)	67.6	75.6	56.7	*χ*^2^(1,*N* = 67) = 0.819; n.s.
Diploma or university (%)	26.8	24.4	30.0	
No response (%)	5.6	0.0	13.3	
Marital (partnership) status				
In a relationship (%)	57.8	70.7	56.7	*χ*^2^(1,*N* = 69) = 6.752; **
Not in relationship (%)	39.4	26.8	40.0	
No response (%)	2.8	2.5	3.3	
Employmalet (or study)				
Employed or studying (%)	54.9	46.3	66.7	*χ*^2^(1,*N* = 61) = 2.160; n.s.
Unemployed (%)	31.0	36.6	23.3	
No response (%)	14.1	17.1	10.0	
Beginning of deafness				
Pre-lingual (%)	36.6	34.1	40.0	*χ*^2^(1,*N* = 65) = 0.041; n.s.
Post-lingual (%)	54.9	53.7	56.7	
No response (%)	8.5	12.2	3.3	
Device used				
CI	32	18	15	*χ*^2^(1,*N* = 71) = 0.258; n.s.
CI + HA	39	23	15	
CI experience (years)				
Mean (SD)	4.08 (2.51)	4.08 (2.43)	4.08 (2.69)	*U* = 441.5; n.s.
Range	1–10	1–9	1–10	
No response (%)	14%	12%	17%	
Age at CI (years)				
Mean (SD)	38.57 (12.09)	40.02 (11.62)	36.48 (12.66)	*U *= 377.5; n.s.
Range	18–57	18–56	19–57	
sat-VAS (%)				
Mean (SD)	78.89 (21.87)	77.6 (24.49)	80.66 (17.93)	*U* = 601.0; n.s.
Range	1.24–100	1.24–100	42.86–100	

*n.s.* not significant

*p < 0.05; **p < 0.01

### Design

The research was conducted by mail. Respondents received a letter requesting anonymous and voluntary participation, questionnaires for assessment of CI benefits and mental distress, and a survey form. In total, responses were received from 71 persons (a response rate of 64%). All procedures were approved by the ethics committee of the Institute of Physiology and Pathology of Hearing, Warsaw (approval number IFPS:/KB/02/2014). No written consent was provided, since the study was based on anonymous questionnaires.

### Subjective assessment of CI benefits and satisfaction

#### Nijmegen Cochlear Implant Questionnaire (NCIQ)

This tool by Hinderink et al. [6] consists of 60 items and is used to probe the benefits of a CI in three areas: physical functioning, with subdomains of “basic sound perception” (NCIQ1), “advanced sound perception” (NCIQ2), and “speech production” (NCIQ3); psychological functioning, with subdomain “self-esteem” (NCIQ4); and social functioning, with subdomains “activity limitation” (NCIQ5) and “social interaction” (NCIQ6). The results were calculated for each scale according to a recently introduced key [32] and were converted to a scale from 1 to 100 using the algorithm provided. The higher the total number of points, the greater the benefits for the CI user in the given area. The NCIQ total score, which is the average of all 6 scales, was also calculated; it is not provided by the creators of the tool, but is used in many works to determine the “average level of CI benefits in a given group of users of this device” [9–11, 14].

NCIQ was translated into Polish by native (bilingual) translators using the forward–backward translation procedure, similar to some earlier studies [9]. The final language version of NCIQ was verified (in terms of its adequacy for PD patients) by a team of three competent judges (2 speech therapists, 1 psychologist) who together had many years of clinical experience in working with deaf and hearing-impaired people (DHH).

#### Satisfaction with a cochlear implant (Sat-VAS)

The subject was asked to indicate, on a scale of 1–10, the extent to which they were satisfied with their cochlear implant. They indicated their degree of satisfaction by placing a point somewhere on a line 161 mm long, with its two ends marked 1 (“I am very dissatisfied”) and 10 (“I am very satisfied”). The results obtained in this way were calculated as a percentage [7].

### Mental distress

#### General Health Questionnaire

(GHQ-28) is used as a screening tool to assess the mental health of adults. It contains 28 items that relate to conditions over the last few weeks. There are four scales: Scale A, somatic symptoms; Scale B, anxiety and insomnia symptoms; Scale C, social dysfunction symptoms; and Scale D, depression symptoms. A Likert method of scoring (0–1–2–3) was used, which means that 0–21 points are obtained on each scale. Higher scores mean more intense symptoms. The GHQ-28 questionnaire also gives an overall result, which is the total points obtained on all scales and indicates the level of overall psychological distress. The official Polish adaptation of GHQ-28 was used, a test aimed at the working population of 18–65 years of age [33].

### Survey form

The general information questionnaire included questions about sociodemographic variables, as well as those related to the PD and CI of the subjects, along with a question about CI satisfaction.

### Statistical analysis

Statistical analysis was performed using the SPSS statistics package version 16 using the following tests: Pearson *χ*^2^, Student’s *t* test, Mann–Whitney *U* test, and Pearson correlation coefficient. As a criterion of significance, a 95% confidence level (*p* < 0.05) was chosen. Because CI satisfaction exhibited a ceiling (due to a very high number of positive results) and did not have a normal distribution, it was logarithmically transformed before calculating correlations.

## Results

Satisfaction with the CI (Sat-VAS) was on average 78% (Table 1) and was similar for both males and females.

The results showing the benefits of a CI (NCIQ) are presented in Table 2. The greatest benefits experienced by PD CI users were in the spheres of “social interaction” (NCIQ 5),”activity limitation” (NCIQ 6), and also in the physical domain, i.e., in “basic sound perception” (NCIQ 1) and were similar for both females and males. However, females experienced significantly less benefits of CI in the psychological sphere of “self-esteem” (NCIQ 4) and in the field of “activity limitation” (NCIQ 6).

**Table 2:** Means and standard deviations of NCIQ results in PD CI users

NCIQ (1–100)	All participants	Female	Male	Statistical test (female vs male)
NCIQ 1—basic sound perception	69.23 (17.83)	67.25 (18.70)	71.95 (16.48)	*U* = 546.5; n.s.
NCIQ 2—advanced sound perception	59.92 (18.87)	58.38 (17.84)	62.02 (20.32)	*t*(69) = 0.798; n.s.
NCIQ 3—speech production	66.9 (20.98)	63.92 (20.35)	71.02 (21.49)	*t*(67) = 1.397; n.s.
NCIQ 4—self-esteem	59.99 (21.89)	54.35 (22.64)	65.56 (19.28)	*U* = 400.0*
NCIQ 5—activity limitation	70.58 (19.99)	66.20 (21.36)	76.59 (16.45)	*U* = 442.5*
NCIQ 6—social interaction	71.53 (16.09)	68.75 (16.24)	75.25 (15.38)	*U* = 439.5; n.s.
NCIQ total	66.17 (16.27)	63.16 (16.48)	70.27 (15.30)	*t*(69) = 1.85; n.s.

*n.s.* not significant

*p < 0.05

In the whole group of respondents, mental distress (GHQ-28 Total) was higher than in the general population of working people, as was the severity of symptoms obtained on the B, C, and D scales of the GHQ-28 questionnaire (Table 3). In terms of gender, females had higher values of GHQ-28 Total, as well as on all of its scales A, B, C, and D compared to the general population; whereas for males, it remained at the same general level. At the same time, females experienced a higher level of anxiety and insomnia (B scale) compared to males.

**Table 3:** Means and standard deviations for results of mental distress (total GHQ-28 and scales A, B, C, D) in people with partial deafness, and comparison between female and male and with the general population (standards from Makowska and Merecz [33])

GHQ-28	All participants	Female	Male	Standards	Statistical test			
					All vs standards	Female vs standards	Male vs standards	Female vs male
Scale A	8.31 (4.43)	9.14 (4.05)	7.16 (4.75)	7.8 (4.52)	*t*(70) = 0.968; n.s.	*t*(40) = 2.127*	*t*(29) = − 0.73; n.s.	*t*(69) = 1.89; n.s.
Scale B	8.66 (5.05)	10.12 (4.75)	6.67 (4.682)	7.29 (4.87)	*t*(70) = 2.289; *	*t*(40) = 3.814**	*t*(29) = − 0.708; n.s.	*U* = 375.0**
Scale C	9.52 (4.14)	9.61 (4.27)	9.40 (4.03)	7.96 (2.81)	*t*(70) = 3.172; **	*t*(40) = 2.47*	*t*(29) = 1.957; n.s.	*U* = 597.5; n.s.
Scale D	5.01 (4.70)	5.76 (5.09)	4.00 (3.97)	3.07 (3.75)	*t*(70) = 3.485; **	*t*(40) = 3.38**	*t*(29) = 1.282; n.s.	*U* = 490.5; n.s.
Total	31.51 (16.26)	34.63 (15.80)	27.23 (16.15)	26.12 (12.92)	*t*(70) = 2.792; **	*t*(40) = 3.449**	*t*(29) = 0.378; n.s.	*U* = 453.0; n.s.

*n.s.* not significant

*p < 0.05; **p < 0.01

The correlations of NCIQ with GHQ-28, Sat-VAS, and other factors are set out in Table 4. For the whole group, NCIQ 4, 5, 6, and total correlated negatively with GHQ-28 Total and scales B and D. For females, there were similar correlations with an additional correlation between NCIQ 6 and GHQ-28 scale C. For males, there were no significant correlations between NCIQ and GHQ-28.

**Table 4:** Correlation between CI benefits (NCIQ) and satisfaction (sat-VAS) and mental distress (GHQ-28) and also with CI duration and age at CI implantation in persons with partial deafness (male and female)

	GHQ-28	GHQ-28	GHQ-28	GHQ-28	GHQ-28	sat-VAS	Age	CI experience	Age at CI
	Total	Scale A	Scale B	Scale C	Scale D				
NCIQ 1—basic sound perception									
All	n.s.	n.s.	n.s.	n.s.	n.s.	0.27*	n.s.	n.s.	n.s.
Female	n.s.	n.s.	n.s.	n.s.	n.s.	0.31*	n.s.	n.s.	n.s.
Male	n.s.	n.s.	n.s.	n.s.	n.s.	n.s.	n.s.	n.s.	n.s.
NCIQ 2—advanced sound perception									
All	n.s.	n.s.	n.s.	n.s.	n.s.	n.s.	n.s.	n.s.	n.s.
Female	n.s.	n.s.	− 0.35*	n.s.	n.s.	n.s.	n.s.	n.s.	n.s.
Male	n.s.	n.s.	n.s.	n.s.	n.s.	n.s.	n.s.	n.s.	n.s.
NCIQ 3—speech production									
All	n.s.	n.s.	n.s.	n.s.	n.s.	n.s.	n.s.	n.s.	n.s.
Female	n.s.	n.s.	n.s.	n.s.	n.s.	n.s.	n.s.	n.s.	n.s.
Male	n.s.	n.s.	n.s.	n.s.	n.s.	n.s.	n.s.	n.s.	n.s.
NCIQ 4—self-esteem									
All	− 0.28*	n.s.	− 0.32*	n.s.	− 0.31**	n.s.	n.s.	n.s.	n.s.
Female	n.s.	n.s.	− 0.35*	n.s.	n.s.	n.s.	n.s.	n.s.	n.s.
Male	n.s.	n.s.	n.s.	n.s.	n.s.	n.s.	0.45*	n.s.	0.55**
NCIQ 5—activity limitation									
All	− 0.3**	n.s.	− 0.32**	n.s.	− 0.37**	0.25*	n.s.	n.s.	n.s.
Female	− 0.44**	n.s.	− 0.42**	n.s.	− 0.47**	n.s.	n.s.	n.s.	n.s.
Male	n.s.	n.s.	n.s.	n.s.	n.s.	n.s.	n.s.	n.s.	n.s.
NCIQ 6—social interaction									
All	− 0.28*	n.s.	− 0.29*	n.s.	− 0.36**	n.s.	n.s.	n.s.	n.s.
Female	− 0.45**	n.s.	− 0.42**	− 0.39*	− 0.48**	n.s.	n.s.	n.s.	n.s.
Male	n.s.	n.s.	n.s.	n.s.	n.s.	n.s.	0.42*	n.s.	0.52**
NCIQ total									
All	− 0.24*	n.s.	− 0.28*	n.s.	− 0.31**	n.s.	n.s.	n.s.	n.s.
Female	− 0.33*	n.s.	− 0.39*	n.s.	− 0.35*	n.s.	n.s.	n.s.	n.s.
Male	n.s.	n.s.	n.s.	n.s.	n.s.	n.s.	n.s.	n.s.	n.s.

*n.s.* not significant

*p < 0.05; **p < 0.01

For the entire group, there was no significant relationship between the age and NCIQ and CI satisfaction. Interestingly, for males, there was a significant positive correlation between age (and age at CI) and NCIQ 4 and 6. It was also found that greater CI satisfaction was associated with the perception of greater benefits in the subdomains NCIQ 1 and 6. When taking gender into account, only in females, there was a positive correlation between CI satisfaction and NCIQ 1. Additionally, CI satisfaction was not correlated with age, the period of CI use, or the age at implantation (not shown in Table 4).

## Discussion

The results indicate that the subjective assessment of the benefits of a CI by participants with PD who have used their device for an average of 4 years is strongly associated with their mental status, i.e., their level of experienced mental distress, especially for females. This issue is very rarely raised when analyzing the perception of benefits of CIs in users from various clinical groups. There are currently no studies that have indicated a role for gender in such a relationship.

Patients with PD, who are one important group of CI users, gain the greatest benefits from a CI within the social spheres of “social interaction” (NCIQ 6), “activity limitation” (NCIQ 5), and “basic sound perception” (NCIQ 1), true for both females and males. These results differ from those obtained in other studies (which have used the same tool but considered only post-lingually deaf subjects), where the greatest benefits conferred by a CI were found in the sphere of “speech production” (NCIQ 3) [12]. This may relate to the special difficulties persons with PD have: the most severe consequences of their hearing loss are felt in the area of speech understanding (“I hear but don’t understand”), which obviously results in social difficulties [4, 24, 34]. This implies that the most important benefits of a CI for people with PD are in this sphere. In addition, females with PD perceived less benefit of a CI (compared to males) in the area of “self-esteem” (NCIQ 4). This might be related to their global self-esteem, which is lower than males among the population of DHH CI users [35].

Self-esteem is primarily conditioned by psychosocial factors [36], and so it is difficult to expect that a CI will, in itself, significantly increase it, even after several years of use. Females with PD also perceive less benefit than males in the sphere of “activity limitation” (NCIQ 5). This could be related to difficulties in the sphere of mental health: greater mental distress compared to males with PD and compared to the general population. No relationship was found between NCIQ results and age (and age at CI) for the whole study group, similar to other studies [9]. However, there was a positive relationship in males for the spheres of “self-esteem” (NCIQ 4) and “social interaction” (NCIQ 6); this suggests that younger males in particular find it more difficult to deal with the consequences of hearing loss in these spheres (and adapt to their CI), and requires further research.

There was also no relationship between the years of CI use and NCIQ results, and here, similar results have been obtained by others [10, 14].

Satisfaction with a CI in subjects with PD is similar to that in other CI users (on average 79% [35], independent of gender), and this factor has not been shown to be associated with age at the time of CI, or period of CI use—in contrast with another clinical group, post-lingually deaf CI users [7]. Satisfaction with a CI in the group of subjects with PD is mainly associated with the perception of benefits in the areas of “basic sound perception” (NCIQ 1) and “activity limitation” (NCIQ 5). It should be noted that in males with PD, satisfaction with a CI is not associated either with the perception of CI benefit (NCIQ) or with the level of mental distress.

Total mental distress (GHQ-28) turned out to be higher in PD patients with a CI compared to the general population, and with it a greater severity of symptoms of anxiety, insomnia, depression, and difficulties in social functioning were found. This is consistent with the results of other studies on this clinical group (PD patients without a CI) [24], as well as in the population of DHH people, among whom there are also certainly those with PD [20, 23, 26]. Taking into account the gender of subjects with PD, it was found that only females had an increased level of mental distress in comparison to the general population, and they also had increased levels of anxiety and insomnia relative to males. These results are consistent with research on mental distress in DHH subjects [19] and in the general population [27]. Attention should be drawn to the fact that elevated levels of distress persist despite implantation in females with PD. Compared to other CI users (males), who are at the same level as the general population when it comes to mental distress [7, 28], females require special attention and care for mental health. When dealing with PD CI users, clinicians should strongly consider offering dedicated psychological intervention in order to better deal with stress.

An important finding is the role of gender. We found that, in females with PD and a CI, their psychological state (as assessed by GHQ-28) correlates well with the subjectively assessed benefits of their CI in the psychosocial sphere (NCIQ 4–6). However, this relationship does not apply to males with PD. Only in females, there was a greater level of anxiety and insomnia and tendency to perceive smaller CI benefits in the sphere of “advanced sound perception” (NCIQ 2). These relationships can be bidirectional: experiencing increased symptoms of mental distress is not conducive to perceiving the benefits of a CI or using it optimally; on the other hand, it is possible that females with PD find that auditory training after CI implantation more difficult compared to males [34].

Other researchers [37] have seen, in post-lingually deaf CI users aged over 70 years, a relationship between depression and anxiety and the perception of CI benefits (NCIQ) in all spheres covering physical, psychological, and social function. One can, therefore, assume that the relationship between the subjectively assessed benefits of a CI and psychological distress differs depending on, among other factors, the age of the respondent [37]. Other factors might be the type of deafness (pre/post-lingual) and, as the results here indicate, the degree of hearing loss (profound hearing loss or PD).

Regardless of gender, we find no relationship between satisfaction with a CI and mental distress, including symptoms of anxiety, insomnia, and depression. This finding is opposite to our previous study in a group of post-lingually deaf CI users, where we found that the severity of depression correlated with the level of satisfaction with the CI [7]. The difference might depend on special clinical factors associated with the groups.

This research is a preliminary exploration of an important issue, previously unrecognized: subjective measurement of the benefits of CIs in PD patients. A limitation of the work is that its anonymous design, implemented so that the information collected was as reliable as possible. However, it also meant that the audiological and psychological outcomes could not be directly related. When dealing with CI cases, a delicate issue is that many patients might be reluctant to provide negative responses; they know the whole procedure is expensive and that their future well-being depends on the continued care given them by audiology specialists. Despite the limitations, the results highlight the important role that psychological distress plays in how well a CI is rated, especially by females with PD. This finding has profound implications for clinicians, who every day are called on to rehabilitate the hearing and speech of patients with CIs. Further research will need to refine this psychological aspect, taking into account different groups of CI users and their audiological profiles.

## Conclusions

Our research indicates that the hearing-related quality of life of PD CI users depends not only on the benefits and general satisfaction with the CI device, but also on an associated level of background mental distress. The severity of the distress plays an important role in the user’s assessment of how well the CI works. Distress can hinder the optimal use of the CI, and therefore successful adaptation to the device. In the general sphere of mental health, the relatively poor functioning of PD CI users compared to the general population (as shown by our measures of quite severe mental distress), calls for the professional attention of clinicians. Offering PD CI users some form of psychological intervention, especially for females, is strongly recommended.

## References

[1] Contrera KJ, Betz J, Li L (2016). Quality of life after intervention with a cochlear implant or hearing aid. Laryngoscope.

[2] Loeffler C, Aschendorff A, Burger T (2010). Quality of life measurements after cochlear implantation. Open Otorhinolaryngol J.

[3] Ou H, Dunn CC, Bentler RA, Zhang X (2008). Measuring cochlear implant satisfaction in postlingually deafened adults with the SADL inventory. J Am Acad Audiol.

[4] Kobosko J, Pankowska A, Olszewski L (2017). Subjective and objective assessment of cochlear implant benefit in adults with the prelingual onset partial deafness [in Polish]. Now Audiofonol.

[5] Lloyd H, Jenkinson C, Hadi M (2014). Patient reports of the outcomes of treatment: a structured review of approaches. Health Qual Life Outcomes.

[6] Hinderink JB, Krabbe PF, Van Den Broek P (2000). Development and application of a health-related quality-of-life instrument for adults with cochlear implants: the Nijmegen Cochlear Implant Questionnaire. Otolaryngol Head Neck Surg.

[7] Kobosko J, Jedrzejczak WW, Pilka E (2015). Satisfaction with cochlear implants in postlingually deaf adults and its nonaudiological predictors: psychological distress, coping strategies, and self-esteem. Ear Hear.

[8] Straatman LV, Huinck WJ, Langereis MC (2014). Cochlear implantation in late-implanted prelingually deafened adults: changes in quality of life. Otol Neurotol.

[9] Hirschfelder A, Grabel S, Olze H (2008). The impact of cochlear implantation on quality of life: the role of audiologic performance and variables. Otolaryngol Head Neck Surg.

[10] Le Roux T, Vinck B, Butler I (2017). Predictors of health-related quality of life in adult cochlear implant recipients in South Africa. Int J Audiol.

[11] Häußler SM, Knopke S, Wiltner P (2019). Long-term benefit of unilateral cochlear implantation on quality of life and speech perception in bilaterally deafened patients. Otol Neurotol.

[12] Muigg F, Bliem H, Holzner B (2019). Do personality factors assessed before cochlear implantation predict hearing-related quality of life after cochlear implantation in postlingually deafened adults?. Ear Hear.

[13] McRackan TR, Bauschard M, Hatch JL (2018). Meta-analysis of quality-of-life improvement after cochlear implantation and associations with speech recognition abilities. Laryngoscope.

[14] Capretta NR, Moberly AC (2016). Does quality of life depend on speech recognition performance for adult cochlear implant users?. Laryngoscope.

[15] Skarzynski H, Lorens A, Piotrowska A (2003). A new method of partial deafness treatment. Med Sci Monit.

[16] du Feu M, du Feu M, Chovaz C (2014). Deafened people. Mental health and deafness.

[17] Carlsson PI, Hjaldahl J, Magnuson A (2015). Severe to profound hearing impairment: quality of life, psychosocial consequences and audiological rehabilitation. Disabil Rehabil.

[18] Manchaiah V, Stein G, Danermark B (2015). Positive, neutral, and negative connotations associated with social representation of’ ‘hearing loss’ and ‘hearing aids’. J Audiol Otol.

[19] de Graaf R, Bijl RV (2002). Determinants of mental distress in adults with a severe auditory impairment: differences between prelingual and postlingual deafness. Psychosom Med.

[20] Fellinger J, Holzinger D, Pollard R (2012). Mental health of deaf people. Lancet.

[21] Shin MS, Song JJ, Han KH (2015). The effect of psychosocial factors on outcomes of cochlear implantation. Acta Otolaryngol.

[22] Li CM, Zhang X, Hoffman HJ (2014). Hearing impairment associated with depression in US adults. National Health and Nutrition Examination Survey 2005–2010. JAMA Otolaryngol Head Neck Surg.

[23] Fellinger J, Holzinger D, Gerich J (2007). Mental distress and quality of life in the hard of hearing. Acta Psychiatr Scand.

[24] Ciesla K, Lewandowska M, Skarżyński H (2016). Health-related quality of life and mental distress in patients with partial deafness: preliminary findings. Eur Arch Otorhinolaryngol.

[25] Kim SY, Kim H-J, Park E-K (2017). Severe hearing impairment and risk of depression: a national cohort study. PLoS ONE.

[26] Kvam MH, Loeb M, Tambs K (2007). Mental health in deaf adults: symptoms of anxiety and depression among hearing and deaf individuals. J Deaf Stud Deaf Educ.

[27] Williams LJ, Berk M, Henry MJ (2014). Depression following fracture in women: a study of age-matched cohorts. BMJ Open.

[28] Bosdriesz JR, Stam M, Smits C (2018). Psychosocial health of cochlear implant users compared to that of adults with and without hearing aids: results of a nationwide cohort study. Clin Otolaryngol.

[29] Brüggemann P, Szczepek AJ, Klee K (2017). In patients undergoing cochlear implantation, psychological burden affects tinnitus and the overall outcome of auditory rehabilitation. Front Hum Neurosci.

[30] Skarzynski H, van de Heyning P, Agrawal S (2013). Towards a consensus on a hearing preservation classification system. Acta Otolaryngol Suppl.

[31] Pruszewicz A, Demenko G, Richter L (1994). New articulation lists for speech audiometry. Part II [in Polish]. Otolaryngol Pol.

[32] Hinderink JB, Krabbe PF, Van Den Broek P (2017). Corrigendum. Otolaryngol Head Neck Surg.

[33] Makowska Z, Merecz D, Makowska Z, Merecz D (2001). Polish adaptation of Goldberg’s general health questionnaires GHQ-12 and GHQ-28. Mental health assessment using Goldberg’s questionnaires, Part 2 [in Polish].

[34] Solnica J, Kobosko J, Pankowska A (2012). Effectiveness of the auditory training in patients with the partial deafness after cochlear implantation in the assessment of patients and speech therapists [in Polish]. Now Audiofonol.

[35] Kobosko J, Jędrzejczak WW, Gos E (2018). Self-esteem in the deaf who have become cochlear implant users as adults. PLoS ONE.

[36] Orth U (2018). The family environment in early childhood has a long-term effect on self-esteem: a longitudinal study from birth to age 27 years. J Pers Soc Psychol.

[37] Knopke S, Häussler S, Gräbel S (2019). Age-dependent psychological factors influencing the outcome of cochlear implantation in elderly patients. Otol Neurotol.

